# A strategy for selective screening of dual-target bioactive compounds against hypertrophic scar through inhibiting angiotensin II type 1 receptor while stimulating type 2 receptor from Chinese herbs

**DOI:** 10.1186/s13020-025-01065-6

**Published:** 2025-01-27

**Authors:** Lejing Qu, Meizhi Jiao, Zilong Zhang, Yuanyuan Ou, Xue Zhao, Yajun Zhang, Xinfeng Zhao

**Affiliations:** 1 Key Laboratory of Resource Biology and Biotechnology in Western China, Ministry of Education, College of Life Sciences, Northwest University, Xi’an, 710069 China; 2 The Second Affiliated Hospital of Xi’an Medical University, Xi’an Medical University, Xi’an, China

**Keywords:** Rhei Radix et Rhizoma, Drug discovery, Multi-target drugs, Hypertrophic scar, Angiotensin receptor, Immobilized receptor

## Abstract

**Background:**

Cutaneous hypertrophic scar is a fibro-proliferative hard-curing disease. Recent studies have proved that antagonists of angiotensin II type 1 receptor (AT_1_R) and agonists of type 2 receptor (AT_2_R) were able to relieve hypertrophic scar. Therefore, establishing new methods to pursue dual-target lead compounds from Chinese herbs is in much demand for treating scar.

**Methods:**

To this end, we immobilized AT_1_R or AT_2_R onto the surface of silica gel from cell lysates through a specific covalent bond by bioorthogonal chemistry. The columns containing immobilized AT_1_R or AT_2_R were jointly utilized to pursue potential bioactive compounds simultaneously binding to AT_1_R and AT_2_R from the extract of Rhei Radix et Rhizoma. Their functions on AT_1_R and AT_2_R expressions were investigated by in vitro and in vivo experiments.

**Results:**

Aloe-emodin and emodin were identified as the potential bioactive compounds binding to both of the two receptors, thereby improving the appearance and pathomorphology of hypertrophic scar. They blocked the AT_1_R pathway to down-regulate the expression of transforming growth factor-β1 (TGF-β1) and stimulate matrix metalloproteinase-1 (MMP-1) expression. As such, the expression of collagen I/III reduced. Conversely, the bindings of the two compounds to AT_2_R reduced the production of nuclear factor-кB1 (NF-кB1), whereby the generation of interleukin-6 (IL-6) was blocked.

**Conclusion:**

We reasoned that aloe-emodin and emodin had the potential to become dual-target candidates against hypertrophic scar through the regulation of AT_1_R and AT_2_R signaling pathways. It showed considerable potential to become a universal strategy for pursuing multi-target bioactive compounds from Chinese herbs by the utilization of diverse immobilized receptors in a desired order.

**Supplementary Information:**

The online version contains supplementary material available at 10.1186/s13020-025-01065-6.

## Introduction

Cutaneous hypertrophic scar includes physical trauma, surgical incisions, and skin piercings. Suffering from such symptoms, life quality of over 100 million people is significantly impaired annually [1]. Current interventions including intralesional injection of corticosteroids and laser treatment are far from adequate since the exceedingly complex pathogenesis of hypertrophic scar [2, 3]. Recent studies have proved that antagonists (e.g., losartan) of angiotensin II type 1 receptor (AT_1_R) and agonists (e.g., compound 21) of type 2 receptor (AT_2_R) are able to relieve hypertrophic scar [4, 5]. Other reports have also confirmed that the specific expressions of the two receptors often occur in adult scars. These pieces of evidence shift the paradigm in searching for drugs simultaneously binding to AT_1_R and AT_2_R as they are becoming new targets for treating hypertrophic scar [6, 7].

Chinese herbs have been used for fighting hypertrophic scar with a long history in Eastern countries [8, 9]. This provides an alternative resource for searching bioactive compounds against the ailment through diverse mechanisms, including the regulation of AT_1_R and AT_2_R pathways. Nevertheless, the pursuit of new drugs from traditional Chinese medicine remains a great challenge since it is often applied as a mixture. Besides the high throughput screening assays on cellular level, high-performance affinity chromatography has emerged as a powerful alternative for pursuing bioactive substances from traditional Chinese medicine owing to the specific separation capacity. Typical cases include the synthesis of immobilized α_1_-, β_1_-, and β_2_-adrenoceptors [10–12], M_3_ muscarinic acetylcholine receptor (M_3_R) [13], and endothelin A receptor (ET_A_R) [14] stationary phases in natural product analysis. Despite the successful applications, these approaches were limited to single-target interventions. However, the therapeutic efficacy of single-target drugs is frequently suboptimal for diseases involving multiple mechanisms as they commonly intervened a biased target amongst. Simple combinational use of them is theoretically viable to enhance the efficiency, however, it is prone to toxic side effects. There is a desperate need to screen multi-target drugs from Chinese herbs that own synergistic effects by mediating diverse pathogenic mechanisms through concurrently binding to distinct targets.

Taking inspirations from these reports, this work intended to create a strategy for pursuing dual-target bioactive compounds from Rhei Radix et Rhizoma which had been historically used for the treatment of hypertrophic scar in Chinese clinical practice. To this end, we applied haloalkane dehalogenase as a fusion tag (Halo-tag) at the C-terminus of AT_1_R and AT_2_R to express the two proteins in *Escherichia coli* (*E. coli*) cells. We immobilized Halo-tagged AT_1_R or AT_2_R onto silica gels by the specific covalent reaction between the protein tag and 6-bromohexanoic acid modified on the gel surface. Application of the immobilized receptors in the herbal analysis through a joint way enabled to decipher the anti-scar bioactive compounds by affinity chromatographic method. The results indicated that aloe-emodin and emodin acted as the dual-target bioactive compounds in the herb, which inhibited the AT_1_R signaling pathway, while simultaneously stimulating AT_2_R function to fight hypertrophic scar. This study demonstrated the current strategy was potential to act as an alternative for screening dual-target bioactive compounds from Chinese herbs.

## Materials and methods

### Material and instruments

Macroporous silica gel (250 Å, 5.0 μm) was from Lanzhou Institute of Chemical Physics, Chinese Academy of Sciences (Lanzhou, China). Reference standards of angiotensin II (Ang II), valsartan, losartan, candesartan, salbutamol, atenolol, emodin, aloe-emodin, and chrysophanol were supplied by Macklin Biochemical Co., Ltd (Shanghai, China). Reference standard of PD123319 was purchased from TargetMol (Boston, MA, USA). Rhei Radix et Rhizoma was obtained from Beijing Tongrentang Pharmacy (Xi’an, China).

Analysis of Rhei Radix et Rhizoma extract and the binding interactions were performed on the high-performance liquid chromatography (HPLC) system (LC-2030C 3D Plus, Shimadzu, Japan). The compounds of interest were identified by HPLC-tandem mass spectrometry using Shimadzu LCMS-8045 (Shimadzu, Japan) and confirmed by comparing their mass spectra with the data of the corresponding reference standards.

### Expression and immobilization of AT_1_R and AT_2_R

By a reported method [15], we expressed the Halo-tagged AT_1_R and AT_2_R in the *E. coli* system. Briefly, Halo-tagged AT_1_R or AT_2_R were engineered in the pReceiver-B02 plasmid. Subsequent expressions were performed by incubating the vector for 20 h in *E. coli* BL21 (DE3) at 37 °C using Luria-Bertani (LB) solid medium with ampicillin resistance (100 μg/mL). A single colony was collected and transferred into the LB liquid medium (50 mL with 100 μg/mL ampicillin) for an extra 12 h incubation. The cells with ampicillin resistance were used to scale up the expression in an auto-induction medium (600 mL) by performing the incubation for 10 h. Repeating the protocol three times, we achieved 8.0 g cell pellets by centrifuging the culture (1800 mL) for 20 min. We resuspended the pellets in phosphate buffer (80 mL, 20 mM, pH 7.4) to disrupt the cell membranes by ultrasonic assay. Centrifuging the suspension for 20 min at 12,000 rpm, we collected the supernatant as the cell lysates containing AT_1_R or AT_2_R for further analysis by SDS-PAGE.

We accomplished the immobilization of AT_1_R and AT_2_R by a reported method [16]. Aminopropyl silica gel was obtained using 3-aminopropyltriethoxysilane as a silylating reagent. The subsequent reaction was carried out by adding aminopropyl silica gel (1.0 g, 1.0 eq) into reaction mixture solution obtained by dissolving 6-bromohexanoic acid (33.55 mg, 1.2 eq) and N,N-diisopropyl ethylamine (DIEA, 74 μL, 3.0 eq) in dimethylformamide (DMF, 8.0 mL). After the addition of N,N,N′,N′-tetramethyluronium hexafluorophosphate (HATU, 65.18 mg, 1.2 eq) into the mixture, the acylation reaction was allowed to proceed for 4.0 h at room temperature under a vigorously agitating condition. The activated gel was collected by vacuum filtrating the reaction mixture. The collected gels were thoroughly rinsed and straightforwardly mixed with 100 mL cell lysate containing AT_1_R or AT_2_R to react for 60 min. The immobilized receptors were collected by centrifugation and rinsed three times by phosphate buffer (20 mM, pH 7.4) to remove unreacted or non-specific adsorption proteins. The previously mentioned functionalized stationary phase was then packed into a chromatographic column tube (4.6 × 50 mm) for further investigation.

### Characterization of the immobilized receptors

The immobilized AT_1_R or AT_2_R was characterized using X-ray photoelectron spectroscopy (XPS), immunofluorescence technique, and ligand-receptor interaction analysis. XPS was used to analyze the surface elements of silica gels, aminopropyl silica gels, 6-bromohexanoic acid-coated gels, and the immobilized receptors. Immunofluorescence was used to characterize the antibody-binding activity and specificity of the immobilized receptors to the control stationary phases. This aim was achieved by incubating the control stationary phases including 6-bromohexanoic acid modified gels and the immobilized receptors with their primary antibodies at 4 ℃ overnight. Subsequent incubation with anti-rabbit green fluorescent secondary antibody was carried out for 2 h against the light with the removal of the nonspecific adsorption by tris(hydroxymethyl)aminomethane buffered saline with tween (TBST) washing. Green signals were recorded by a fluorescence microscope after the gels were totally rinsed by TBST.

Ligand-binding specificity was characterized by determining the retention times of control drugs like salbutamol and atenolol (non-specific ligands of AT_1_R and AT_2_R), canonical ligands including valsartan, losartan, and candesartan (specific ligands of AT_1_R), and PD123319 (specific ligand of AT_2_R) as well as frontal analysis determination of their association constants with the receptors. The void time of the chromatographic system was tested using sodium nitrite as it has been widely accepted as a non-retained compound on an immobilized protein column. The mobile phase was 20 mM phosphate buffer (pH 7.4) at a flow rate of 1 mL/min at 25 ℃. Frontal analysis was performed on immobilized AT_1_R column using mobile phases containing diverse concentrations of ligands: 0.01, 0.02, 0.05, 0.1, 0.2, 0.5, 1.0, 2.0, and 5.0 μM for valsartan; 0.01, 0.02, 0.05, 0.1, 0.2, 0.5, 1.0, 2.0, and 5.0 μM for losartan; and 0.01, 0.02, 0.05, 0.1, 0.2, 0.5, 1.0, 2.0, 5.0, and 10.0 μM for candesartan. Likewise, the ligand concentrations were 0.05, 0.1, 0.2, 0.5, 1.0, 2.0, 5.0, and 10.0 μM for PD123319; 0.01, 0.02, 0.05, 0.1, 0.2, 0.5, 1.0, 2.0, 5.0, and 10.0 μM for valsartan; 0.05, 0.1, 0.2, 0.5, 1.0, 2.0, 5.0, 10.0, and 20.0 μM for losartan, when they were applied on the immobilized AT_2_R column. Each concentration was triplicated to acquire the breakthrough curves for calculating the binding parameters by Eq. (1) [17]:

1 $$\frac{1}{{m}_{\text{lapp}}}=\frac{1}{{K}_{\text{A}}{m}_{\text{L}}[C]}+\frac{1}{{m}_{\text{L}}}$$

where *m*_lapp_ is the apparent adsorption amount. *m*_L_ denotes the number of binding sites. *K*_A_ represents the association constant, while [*C*] is the ligand concentration.

### Screening of bioactive compounds from Rhei Radix et Rhizoma

The herbal extract was prepared using the heating-reflux method. Briefly, the herb was dried at 60 ℃ prior to being ground as powder (80 meshes). One gram of the dried powder was suspended in 50 mL methanol for 30 min. Subsequent extraction was performed by refluxing the suspension with a duration of 1.0 h. The extracts were combined and condensed to 25.0 mg/mL by rotary evaporation. The condensed extract was treated with a 0.45 μm nylon membrane before chromatographic analysis on AT_1_R and AT_2_R columns. The mobile phase was 20 mM phosphate buffer at 0.2 mL/min flow rate. The peaks detected at 254 nm with retention times greater than the void time were considered as the potential bioactive compounds.

The retained compounds were collected for additional analysis via HPLC-MS/MS. The separation was carried out on an Agilent Eclipse XDB-C_18_ column (3.5 μm, 2.1 mm × 150 mm). The mobile phase was the mixture of water and methanol with an addition of 0.1% formic acid (V: V) at a flow rate of 0.7 mL/min. For the separation of the retained compounds on the AT_1_R column, the ratio of methanol/water was 75:25 (V: V), while it was adjusted to 85:15 (V: V) for AT_2_R-targeting compound separation. The optimized mass spectrometry conditions included: ionizing mode-negative; drying gas-N_2_; flow rate of drying gas-10.0 L/min; temperature-300 ℃; and mass scan range-*m/z* 50 to 1500 Da. Once the compounds of interest were identified by comparing their mass patterns with the data of the reference standards, frontal analysis was designed to assess their drug-like properties by calculating the binding affinity with AT_1_R and AT_2_R.

### Bioactivity of the screened compounds

Human skin fibroblasts (HSF) were purchased from Shanghai Gaining Biotechnology Co. (Shanghai, China) and cultured in Dulbecco's Modified Eagle (DMEM) media (Servicebio, Wuhan, China) containing 10% fetal bovine serum (Every Green, Zhejiang, China). The HSF cell lines incubated at 37 °C in the atmosphere containing 5% CO_2_ were used for assessing the anti-scar bioactivity of the screened compounds. The pathological cells induced by 100 nM Ang-II were cultured in 6-well dishes for a 12 h incubation prior to being exposed to the positive control drugs and the screened compounds with 20 μM concentration, the dose of the reagents was based on results from pre-experiments. After another 12 h incubation, we harvested the cells in radio immunoprecipitation assay (RIPA) lysis buffer containing phenylmethanesulfonyl fluoride (PSMF) for examining the expression of AT_1_R, AT_2_R, and the cytokines associated with their signaling pathway. The concentration of the total proteins in the cell lysates was determined by a bicinchoninic acid (BCA) kit (Beyotime, Shanghai, China), while the protein and the cytokine expressions were tested by western blot analysis.

### Animal grouping and experimental design

All animal experiments were authorized by the Animal Care and Use Committee of Northwest University in China (No. NWU-AWC-20221104L). Male New Zealand white rabbits (2.5 ± 0.2 kg) were obtained from Chengdu Dossy Experimental Animal Co., Ltd. (SCXK (Chuan) 2019-031). Rabbits were housed in a controlled environment (22 ± 2 °C), and 12 h light/dark cycles with free access to food and tap water.

Hypertrophic scar rabbits were modeled according to a previously reported method [18]. Briefly, a 10-mm biopsy punch was used to make six full-thickness lesions down to bare cartilage on each ear of the rabbits. The wounds were ordered randomly and each was ten millimeters in diameter. We also removed the perichondrium, dermis, and epidermis in each wound under sterile conditions. Before the above surgery, all rabbits were treated with sodium pentobarbital (30 mg/kg) intravenously to alleviate their pains. We covered the wounds with sterile gauze for the 1st day. On the 7th day after the surgery, the scars were randomly separated into 4 groups (n = 12): Group 1 served as the model group and received a daily thin coating of basic ointment; groups 2 and 3 were treated with 5% aloe-emodin ointment and 5% emodin ointment, respectively; group 4 were used as positive control group taking 5% valsartan ointment. The dose of the ointment serving to every scar was 5 mg. We performed the above topical treatment once daily for 4 weeks and unwounded rabbits were gathered as a normal group. Animals were sacrificed postoperative for 35 days. The scar tissue was collected and separated into two halves: one was preserved for 3 days in a 4% paraformaldehyde for histopathological analysis, while the other half was separated with the removal of cartilage for Western blot tests. We homogenized the scars tissues using RIPA-containing PSMF solution and collected the supernatant for protein concentration determination.

### Histological analysis

We fixed the scar tissues with 4% paraformaldehyde for 3 days, embedded them in paraffin, and sectioned them with a dermatome. Hematoxylin-eosin (H&E) staining was used to determine the scar elevation index (SEI) [19]. Photography was carried out under the Nikon SMZ25 microscope system, SEI measurement was performed using NIS-Elements Imaging Software Version 4.50. We also observed the collagen deposition by Masson’s trichrome staining, resulting in the collagen fibers blue, the cytoplasm light pink, and the nucleus dark brown, respectively. We analyzed the band density, thickness, placement, and collagen deposition using light microscopy.

### Western blot analysis

The cell lysates and the tissue lysates were separated by 13% SDS-PAGE to transfer the bands of interest onto nitrocellulose filter (NC) membranes. The blotted membranes were blocked for 60 min with 5% nonfat milk (Sangon, Shanghai, China) at room temperature. The blocked membranes were exposed to the appropriate primary antibodies for AT_1_R (Proteintech, Wuhan, China), AT_2_R (Abcam, Cambridge, UK), matrix metalloproteinase-1 (MMP-1), tissue inhibitors of matrix metalloproteinase-1 (TIMP-1, Immunoway, Plano, TX, USA), TGF-β1, IL-6, NF-κB1 (Sangon, Shanghai, China), collagen I, collagen III, and GAPDH (Servicebio, Wuhan, China) at 4 ℃ followed by overnight incubation. With the removal of nonspecific adsorption by 1 × TBST, we used horseradish peroxidase-conjugated anti-mouse or anti-rabbit (Sangon Biotech, Shanghai, China) secondary antibodies to visualize the specific proteins. The band intensities were quantified by Image J 1.53 k software (National Institutes of Health, USA).

### Statistical analysis

One-way ANOVA followed by Tukey tests were used for data analysis and the data were expressed as mean ± SEM. The experiments were independently repeated in triplicate to confirm the findings. Statistical significance was set at *P* < 0.05.

## Results

### ***Preparation and characterization of the immobilized AT***_***1***_***R and AT***_***2***_***R***

**Fig. 1:**
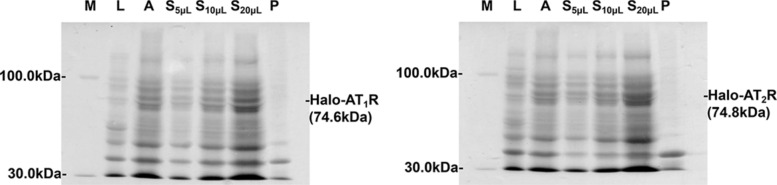
SDS-PAGE analysis of the expression of Halo-AT_1_R and Halo-AT_2_R in *E. coli.* Lane M: protein marker; Lane L: LB medium; Lane A: auto-induction medium; Lane S: supernatant; Lane P: precipitation

The availability of high-quality proteins was a prerequisite for drug discovery. Given this, the development of methods for expressing functional proteins like GPCRs had been propelled in diverse fields. Distinct efforts had thus been made to produce sufficient quantity and quality of GPCRs yet were limited to eukaryotic organisms for native expression of the receptors. Otherwise, such purpose had been sporadically achieved using the *E. coli* system [20]. In this study, AT_1_R and AT_2_R were reconstituted and expressed in *E. coli* cells utilizing Halo-tag as a fusion partner in an auto-induction medium (Table S1). Unlike the LB lane, we observed a new sharp band when the auto-induction medium was applied during the cultures of the two receptors (Fig. 1). Such a band appeared to be much more intensive in the supernatant in comparison with the precipitation. These indicated that the reconstituted proteins were expressed as homogenous and active forms. As such, we intended to identify the band of interest in the two gels by calculating their molecular weights.

The logarithm of molecular weights (M) of the marker bands were linearly related to the mobilities of the bands (x) with a regression equation of lgM = 2.29–0.82x. This equation allowed a calculation of 74.6 kDa for the band of interest (Fig. 1). In such a calculation, we identified the band as Halo-tagged AT_1_R since the theoretical molecular weights of Halo-tag and the free receptor were 35.2 and 39.4 kDa. The relative content of the receptor was measured as 5.57% by grayscale analysis. Upon this result, we reasoned that the level of AT_1_R in the *E. coli.* system was 1.88 mg/L since the total proteins in the culture supernatants were determined to be 33.03 mg/L by the BCA method. Likewise, the band of interest was identified as Halo-tagged AT_2_R with a molecular weight of 74.8 kDa. The expression of the receptor in *E. coli.* was 1.69 mg/L. These results, in summary, demonstrated that the *E. coli.* system was feasible to express soluble AT_1_R and AT_2_R at the milligram level, thereby providing sufficient proteins for subsequent investigations like purification and immobilization.

**Fig. 2:**
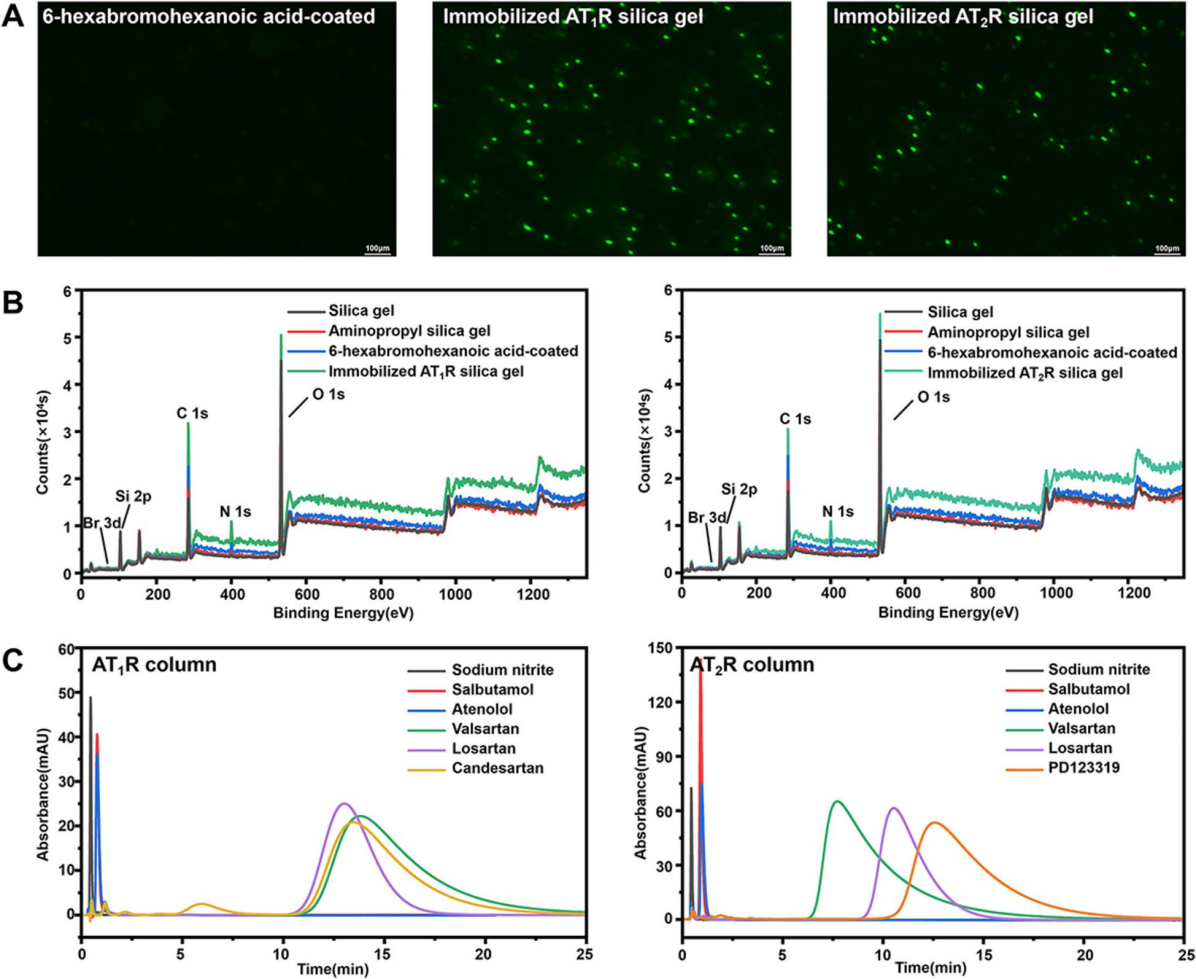
Characterization of immobilized AT_1_R and AT_2_R. **A** Antibody specificity analysis. **B** Surface element analysis. **C** Specificity analysis

We immobilized the abovementioned receptors onto the surface of silica gels based on the bioorthogonal chemistry between the Halo-tag and 6-bromohexanoic acid (Fig. S1). The elemental contents of the immobilized receptor surfaces were examined by XPS (Fig. 2B). The bare gels exhibited observable peaks of O 1s at 532.79 eV, C 1s at 284.8 eV, and Si 2p at 103.35 eV. Introducing aminopropyl onto the gels resulted in a new peak of N 1s at 399.53 eV, which was calculated to be 2.81% of all the elements. Further modification by 6-bromohexanoic acid afforded the gels a demonstrable peak of Br 3d at 68.16 eV with a relative content of 0.2%. Besides, the content of nitrogen was increased to 3.00% owing to the modification. As anticipated, the peak of bromine disappeared after the gels reacted with the fusion AT_1_R and AT_2_R. Conversely, the relative contents of nitrogen changed to 5.84% and 5.58% ascribed to the high density of amino groups in the receptor structures (Table S2). These, taken together, demonstrated the successful immobilization of the two receptors by the desired protocol.

The next step was designed to investigate the binding specificity of the immobilized receptors to their antibodies by fluorescence microscopy. A little fluorescence signal was observed when we treated 6-bromohexanoic acid using the primary antibodies of AT_1_R and AT_2_R and incubated the resulting gels with their anti-rabbit green fluorescent secondary antibodies (Fig. 2A). Conversely, we observed intensive fluorescence adsorptions when it comes to immobilized AT_1_R and AT_2_R under identical conditions. These results indicated that the two receptors were immobilized onto the gels through covalent bonds without non-specific adsorption when the proposed immobilization method and the rinsing protocol were performed. This enabled immobilized AT_1_R and AT_2_R to retain the binding specificity to their primary antibodies.

The capacity of immobilized AT_1_R and AT_2_R to recognize and interact with their specific ligands was investigated by the retention times of canonical drugs on the columns packed with each kind of receptor alone. Using sodium nitrite as a solute, the void time of the chromatographic system was determined to be 0.44 min under the proposed conditions. The canonical drugs including valsartan, losartan, and candesartan presented retention times of 13.82, 13.01, and 13.47 min on the AT_1_R column. The non-subtype specific ligands, valsartan and losartan also exhibited weaker retentions of 7.73 and 10.54 min on the column containing immobilized AT_2_R. While the high-specific ligand PD123319 displayed a retention time of 12.58 min on the same column under identical conditions. Unlike them, the specific ligand of β_2_-adrenoceptor (salbutamol) and the canonical drug of β_1_-adrenoceptor (atenolol) demonstrated the retentions approximated to the void time (Fig. 2C). These findings indicated that immobilized AT_1_R and AT_2_R were capable of binding to the specific ligands, and separated them by their retention times on the column packed with the corresponding receptor.

With these retention characters in hand, we intended to pursue the viability of the immobilized receptor columns in probing drug-receptor binding affinity. This purpose was achieved by examining the interaction between the abovementioned specific ligands and certain receptors by frontal analysis. All six ligands presented typical breakthrough curves with the loss of retentions when the drug concentrations in the mobile phases were increased (Fig. S2A and S3A). This indicated a good line with the theoretical hypothesis of frontal analysis. As such, we exported the raw data of these curves to probe the real adsorption model of the drugs on the columns by adsorption energy distribution (AED) analysis. The unimodal AED profiles demonstrated that the adsorption sites of valsartan, losartan, and candesartan on the immobilized AT_1_R column were evenly distributed (Fig. S2B). Likewise, the same model was suitable for describing the adsorption of PD123319, valsartan, and losartan on the immobilized AT_2_R column (Fig. S3B). Upon these results, we reasoned that one kind of binding site on the AT_1_R and AT_2_R columns dominated the bindings of the ligands to the receptors. This allowed us to calculate their association constants to AT_1_R and AT_2_R by the Langmuir isotherm model (Fig. S2C and S3C). As summarized in Table 1, the association constants of valsartan, losartan, and candesartan to AT_1_R were (1.79 ± 0.07) × 10^6^ M^−1^, (6.21 ± 0.36) × 10^5^ M^−1^ and (1.06 ± 0.05) × 10^6^ M^−1^. The numbers of their binding sites on the column were (1.24 ± 0.04) × 10^–8^ M, (2.15 ± 0.12) × 10^–8^ M, and (7.38 ± 0.38) × 10^–9^ M. The association constants of PD123319, valsartan, and losartan to AT_2_R were (2.11 ± 0.03) × 10^5^ M^−1^, (8.26 ± 0.03) × 10^5^ M^−1^, and (2.06 ± 0.02) × 10^5^ M^−1^ with the numbers of binding sites of (7.44 ± 0.09) × 10^–8^ M, (2.28 ± 0.01) × 10^–8^ M, and (7.37 ± 0.05) × 10^–8^ M. The rank order of the drug association constants to AT_1_R in this work was in good line with the data in the reports where radio-ligand binding assay was applied with losartan [21, 22], thereby confirming the viability of immobilized AT_1_R in probing the drug-receptor affinity interaction. Unlike AT_1_R, the bindings of ligands to AT_2_R were seldom reported except for the case of PD123319. This challenged the comparison of the association constants in current work with that obtained by typical pharmacological assays. When we made a comparison, the association constant of valsartan to AT_1_R was one order of magnitude higher than that of AT_2_R (Table 2). This presented a good agreement with the report that the two drugs were more selective to AT_1_R over AT_2_R [23]. These observations suggested that immobilized AT_2_R was also possible to realize the interaction between the receptor and its specific ligands in terms of their association constants, which provided an alternative for pursuing potential bioactive compounds binding to the receptor from a complex system.

**Table 1:** Binding parameters of AT_1_R to five ligands by frontal analysis on the AT_1_R column (n = 3, mean ± SD)

Ligand	Equation	R^2^	*K*_A_ (M^−1^)	*m*_L_ (M)
Valsartan	$\frac{1}{{m}_{lapp}}=(44.96\pm 0.57)\times \frac{1}{[C]}+(8.11\pm 0.03)\times {10}^{7}$	0.9977	(1.79 ± 0.07) × 10^6^	(1.24 ± 0.04) × 10^–8^
Losartan	$\frac{1}{{m}_{lapp}}=(74.53\pm 0.59)\times \frac{1}{[C]}+(4.67\pm 0.03)\times {10}^{7}$	0.9996	(6.21 ± 0.36) × 10^5^	(2.15 ± 0.12) × 10^–8^
Candesartan	$\frac{1}{{m}_{lapp}}=(126.74\pm 0.55)\times \frac{1}{[C]}+(1.36\pm 0.07)\times {10}^{8}$	0.9997	(1.06 ± 0.05) × 10^6^	(7.38 ± 0.38) × 10^–9^
Aloe-emodin	$\frac{1}{{m}_{lapp}}=(64.65\pm 0.07)\times \frac{1}{[C]}+(1.29\pm 0.01)\times {10}^{7}$	0.9996	(2.00 ± 0.02) × 10^5^	(7.74 ± 0.06) × 10^–8^
Emodin	$\frac{1}{{m}_{lapp}}=(35.30\pm 0.12)\times \frac{1}{[C]}+(1.83\pm 0.07)\times {10}^{7}$	0.9989	(5.19 ± 0.21) × 10^5^	(5.49 ± 0.22) × 10^–8^

**Table 2:** Binding parameters of AT_2_R to five ligands by frontal analysis on the AT_2_R column (n = 3, mean ± SD)

Ligand	Equation	R^2^	*K*_A_ (M^−1^)	*m*_L_ (M)
PD123319	$\frac{1}{{m}_{lapp}}=(63.75\pm 0.16)\times \frac{1}{[C]}+(1.34\pm 0.02)\times {10}^{7}$	0.9994	(2.11 ± 0.03) × 10^5^	(7.44 ± 0.09) × 10^–8^
Valsartan	$\frac{1}{{m}_{lapp}}=(53.21\pm 0.09)\times \frac{1}{[C]}+(4.39\pm 0.02)\times {10}^{7}$	0.9984	(8.26 ± 0.03) × 10^5^	(2.28 ± 0.01) × 10^–8^
Losartan	$\frac{1}{{m}_{lapp}}=(65.90\pm 0.07)\times \frac{1}{[C]}+(1.36\pm 0.01)\times {10}^{7}$	0.9998	(2.06 ± 0.02) × 10^5^	(7.37 ± 0.05) × 10^–8^
Aloe-emodin	$\frac{1}{{m}_{lapp}}=(62.93\pm 0.11)\times \frac{1}{[C]}+(1.55\pm 0.06)\times {10}^{6}$	0.9999	(2.45 ± 0.10) × 10^4^	(6.48 ± 0.27) × 10^–7^
Emodin	$\frac{1}{{m}_{lapp}}=(104.66\pm 0.30)\times \frac{1}{[C]}+(1.60\pm 0.04)\times {10}^{7}$	0.9995	(1.53 ± 0.05) × 10^5^	(6.25 ± 0.18) × 10^–8^

### Aloe-emodin and emodin were dual-target compounds binding to AT_1_R and AT_2_R

**Fig. 3:**
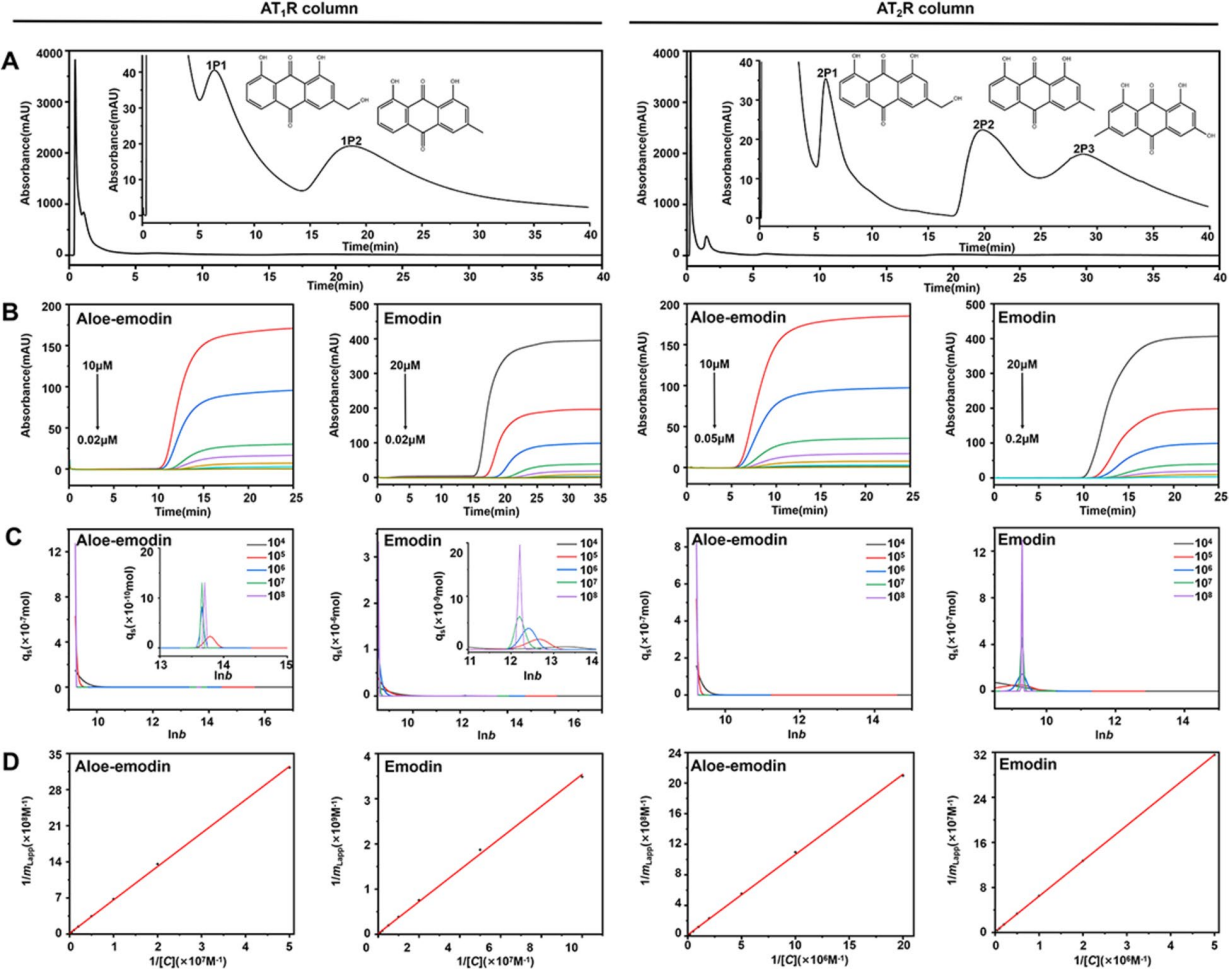
Bioactive compound screening and evaluation from Rhei Radix et Rhizoma. **A** Representative chromatogram of Rhei Radix et Rhizoma extract on AT_1_R and AT_2_R column. **B** Breakthrough curves. **C** AED analysis. **D** Plots of 1/*m*_Lapp_ versus 1/[*C*] for bioactive compounds of Rhei Radix et Rhizoma on the AT_1_R and AT_2_R column

With the immobilized AT_1_R and AT_2_R in hand, we pursued the dual-target bioactive compounds from Rhei Radix et Rhizoma using these two immobilized receptors sequentially. We observed three demonstrable peaks when immobilized AT_1_R was applied as the stationary phase (Fig. 3A). Among them, the first peak presented a retention time without a clear difference from the void time, thus denoting the compounds that had little affinity to the receptor. The other two peaks (1P1 and 1P2) exhibited much longer retention times than the void time, whereby we attributed them to the compounds binding to AT_1_R. Likewise, 2P1, 2P2, and 2P3 were primarily identified as the potential bioactive compounds that interacted with AT_2_R. These attributions were further confirmed by subsequent separation and identification of the five peaks using HPLC-MS/MS. Peaks 1P1 and 2P1 generated the MS/MS pattern of *m/z* 269.0 [M-H]^−^/240.0 [M-H-COH]^−^ (Fig. S4). Regarding such character, we identified the two peaks the same as aloe-emodin since the MS/MS information was in good line with the mass pattern of the reference standard (Fig. S5). By similar analysis, we identified peaks 1P2 and 2P2 as emodin while attributing peak 2P3 as chrysophanol. Therefore, we reasoned that aloe-emodin and emodin severed as the potential dual-target bioactive compounds in Rhei Radix et Rhizoma binding to AT_1_R and AT_2_R as they exhibited demonstratable retentions on the two columns.

### Evaluation of drug-receptor binding affinity

Following the identification of aloe-emodin and emodin, we performed frontal analysis, which was the classical chromatographic method for evaluating the affinity of the drugs binding to receptors (Fig. 3B). First, the two compounds presented homogenous AED profiles when they interacted with AT_1_R and AT_2_R (Fig. 3C). This allowed us to calculate their association constants to the two receptors by the Langmuir isotherm model (Fig. 3D). As summarized in Tables 1 and 2, the association constants of aloe-emodin and emodin to AT_1_R were (2.00 ± 0.02) × 10^5^ M^−1^ and (5.19 ± 0.21) × 10^5^ M^−1^. Such data were much lower than their affinity to AT_2_R on which the association constants were calculated as (2.45 ± 0.10) × 10^4^ M^−1^ and (1.53 ± 0.05) × 10^5^ M^−1^. Remarkably, the association constants of the two compounds to AT_2_R were close to the data of the high specific ligand (PD123319). This implied that aloe-emodin and emodin had the potential to become specific ligands of the receptor, thus having the possibility to be developed as new drugs against the hypertrophic scar.

### Aloe-emodin and emodin exhibited anti-hypertrophic scar activity through AT_1_R and AT_2_R pathway in vitro

**Fig. 4:**
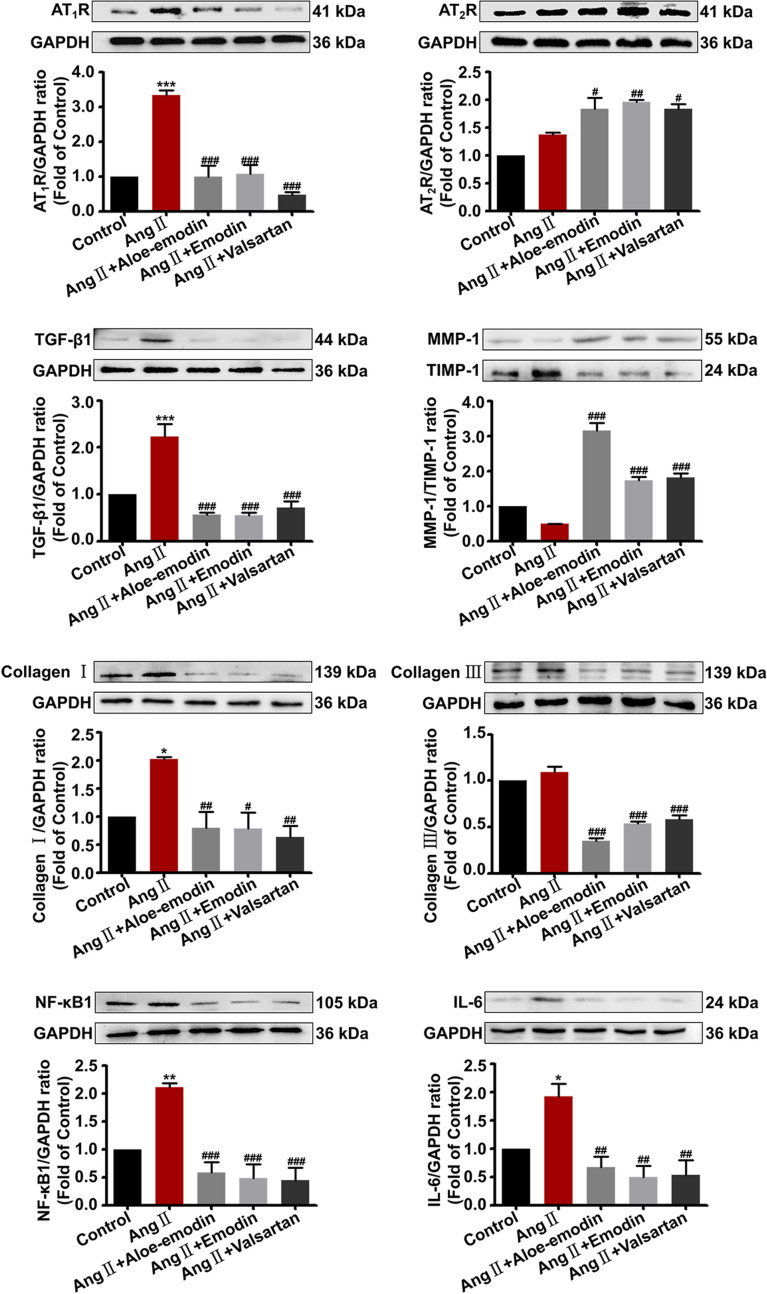
Effects of drugs on the expression of AT_1_R and AT_2_R and their signaling pathway proteins (n = 6). Drug treated group vs. normal group: *P < 0.05, **P < 0.01 and ***P < 0.001; drug treated group vs. the Ang II group: ^#^*P* < 0.05, ^##^*P* < 0.01 and ^###^*P* < 0.001. The detailed statistical analysis was tabulated in Table S3

We evaluated the potential effects of aloe-emodin and emodin on Ang II-induced HSF cells in terms of fibrosis and inflammation. As illustrated in Fig. 4, compared with the model cells induced by 100 nM Ang II, the cells treated with aloe-emodin and emodin demonstrated a lower level of AT_1_R expression. The expressions of IL-6, TGF-β1, TIMP-1, and collagen I/III were substantially reduced, while the level of MMP-1 was increased. These indicated that aloe-emodin and emodin were able to block the function of AT_1_R, thereby blocking hypertrophic scar through the fibrotic pathway. On the contrary, the expression of AT_2_R was clearly increased while the cells were treated with aloe-emodin and emodin. Besides the receptor expression, we observed the two compounds reduced the production of IL-6 as well. The reason might cause the inhibition of NF-кB and the stimulation of interleukin-10 (IL-10) by the two compounds. Upon these results, we deduced that aloe-emodin and emodin demonstrated stimulation effects on AT_2_R to exert anti-scar effect through the inflammatory pathway. Collectively, it was reasoned that aloe-emodin and emodin were potential dual-target bioactive compounds of Rhei Radix et Rhizoma against hypertrophic scar.

### Aloe-emodin and emodin mediating AT_1_R and AT_2_R pathway in vivo

**Fig. 5:**
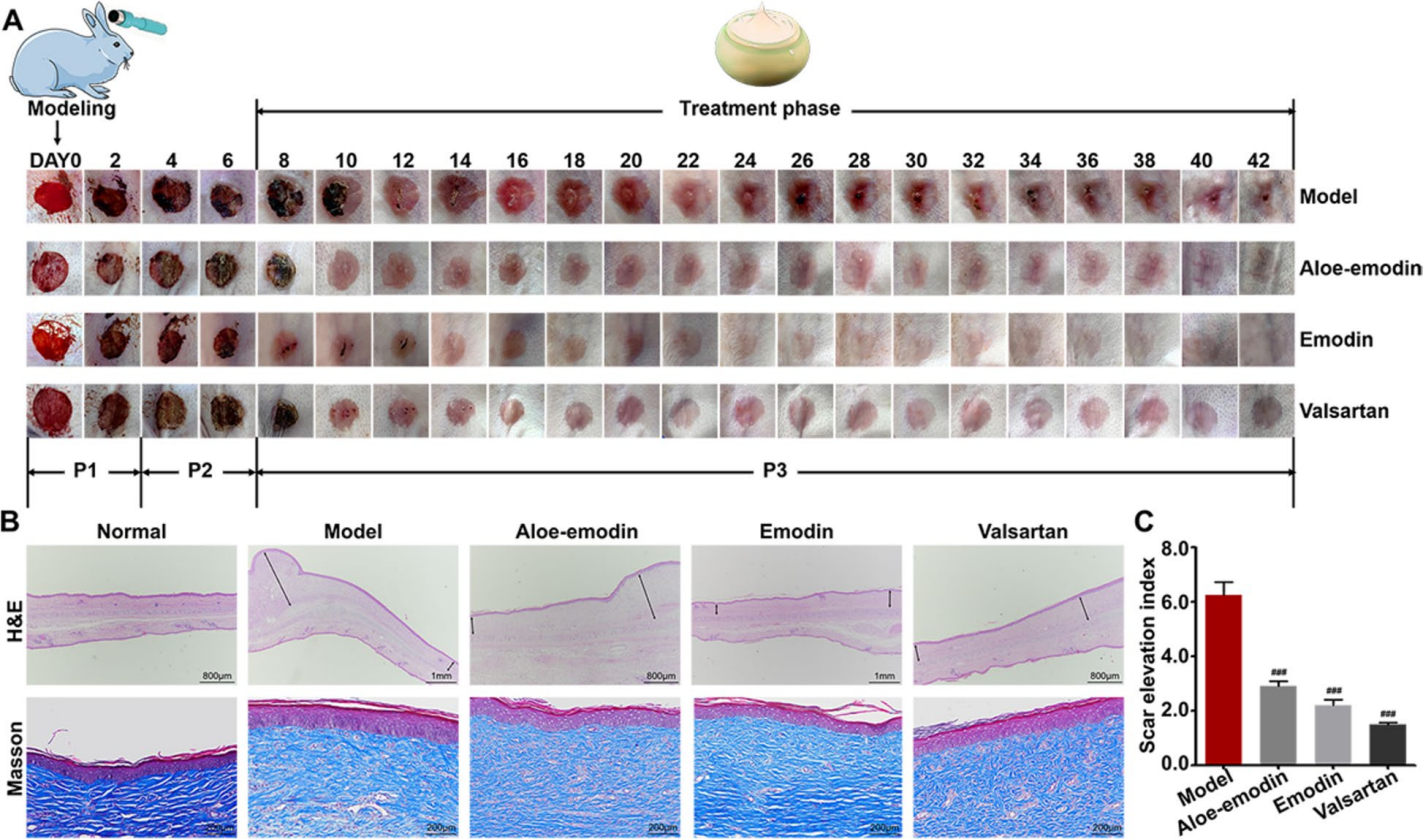
Effect of drugs on the morphology of rabbit ear scar tissue (n = 6). **A** Representative photographs of wound closure evolution for the different treatments by 42 days. **B** H&E and Masson’s trichrome staining analysis of Pathological changes in the scar tissues. **C** SEI of scar tissues. Drug treated group vs. the model group: ^###^*P* < 0.001. P1: Haemostasis and inflammatory phase; P2: Proliferative phase; P3: Remodelling phase

Initially, an assessment was conducted on the scar morphology of the rabbit ear scar model, which was categorized into three distinct aspects. The photographs depicting the progression of wound closure over a period of 42 days revealed that the scar in the model group exhibited a protruding, irregular shape, uneven height, flushed and congested appearance, and a solid and tough texture (Fig. 5A). Conversely, the scars in the three treatment groups displayed a regular shape and relatively flat surface, with the emodin group exhibiting a color closest to that of normal skin, followed by aloe-emodin. Masson’s trichrome staining demonstrated that the scars in the model group exhibited a high degree of thickness, density, and tangled collagen fibers (Fig. 5B). Compared with the model group, the collagen fibers in the three treatment groups were more regularly arranged with relatively fewer disorganized pattern. Among them, the emodin treatment group was uniformly arranged, with thinner and sparser collagen fibers which appeared to be closer to those of normal skin. H&E staining showed that scars in the model group were significantly thickened. Unlike this, SEI in the three treatment groups were significantly lower, of which the valsartan-positive group was the lowest followed by the group treating with emodin (Fig. 5C). Ultimately, the emodin group demonstrated significant improvement in scar color, thickness, and texture, yielding the most favorable outcomes.

**Fig. 6:**
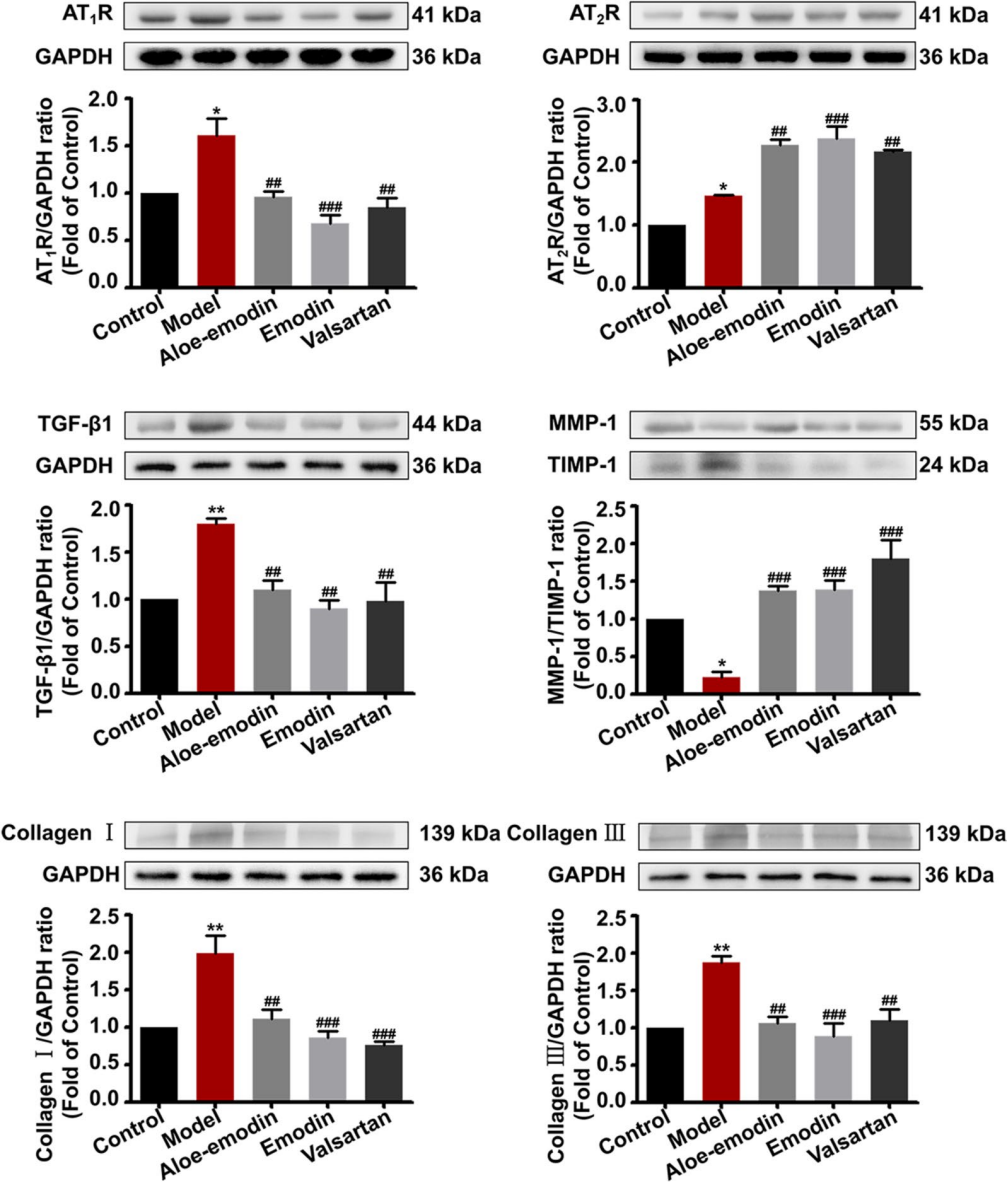
Treatment mechanism of drug on scar tissue in rabbit ears (n = 6). Drug treated group vs. normal group: *P < 0.05 and **P < 0.01; drug treated group vs. the model group: ^##^*P* < 0.01 and ^###^*P* < 0.001. The detailed statistical analysis was tabulated in Table S4

Subsequently, an exploration was undertaken to examine the therapeutic mechanisms associated with both AT_1_R and AT_2_R mediated downstream fibrotic and inflammatory pathways. Compared with the normal group, the AT_1_R, AT_2_R, and TGF-β1 levels in the model group were increased (*P* < 0.05), the MMP-1/TIMP-1 ratio was substantially reduced (*P* < 0.05), resulting in increased collagen I/III levels (*P* < 0.01). Treating the rabbit with aloe-emodin, emodin, and valsartan resulted in the loss of AT_1_R expression and elevated AT_2_R levels. The expressions of TGF-β1, TIMP-1, and collagen I/III were substantially reduced, while the level of MMP-1 was increased (Fig. 6). These indicated that aloe-emodin and emodin had the capacity to down-regulate the expression of AT_1_R as well as to block the function of the receptor, while at the same time stimulated AT_2_R function to fight hypertrophic scar. Among them, the MMP-1/TIMP-1 ratio of the emodin treatment group was more similar to that of normal skin, and their collagen I levels were reduced more significantly (*P* < 0.001). These results demonstrated the feasibility of aloe-emodin and emodin in hypertrophic scar treatment by regulating the AT_1_R and AT_2_R signaling pathways.

## Discussion

Hypertrophic scars were the result of excessive secretion of collagen, proteoglycans, elastin, and extracellular matrix (ECM) during the wound-healing process. Such complex pathological mechanism necessitated the careful selection of an animal model during the study. Typical operations using dorsal skin scars of rats or mice were often applied to observe wound healing while were limited to assessing the scar color and size. Because it was difficult to form a hypertrophic scar when the dorsal skin was wrinkled and healed. For a similar reason, it was not feasible to model human scars by transplanting nude mice. Conversely, the rabbit ear scar provided better observation of scar color, thickness, flatness, and texture. These made it an ideal model for studying the pathologic progress of hypertrophic scar, particularly, when drugs were applied as it. As such, this work preferred to use rabbit ear scars as the animal model since AT_1_R and AT_2_R mainly mediate the progress of hypertrophic scar.

As illustrated in Fig. 5A, the dynamic progress of scar formation included hemostasis and inflammation, proliferation, and remodeling after rabbit ear trauma was operated on [24]. At the initial stage, hemostasis happened due to the aggregation of platelets. As a result, cytokines like IL-6 and TGF-β were released to induce an ordered distribution of distinct cells including inflammatory cells, epithelial cells, and fibroblasts. Owing to the role of these cells, the subsequent proliferation phase started to generate ECM. Finally, remodeling occurred due to the reshaping of the dermal matrix through the release of MMP and collagen fibers from fibroblasts/myofibroblasts. Throughout the abovementioned stages, the inflammatory response was enhanced and the expression of growth factors including TGF-β was up-regulated. These increased ECM expression, thereby making fibroblast overactivation to form an undesirable scar. Taking together, regulating TGF-β expression or balancing pro-inflammatory and anti-inflammatory cytokines (e.g. IL-6 and interleukin-10) played a particular role in treating hypertrophic scar. Likewise, activating collagenase expression and increasing collagen I/III degradation also behaved as alternative ways for fighting hypertrophic scar [25]. As such, target proteins that mediated the abovementioned pathways were particularly attractive to create affinity-based strategies for pursuing bioactive compounds against the hypertrophic scar.

AT_1_R and AT_2_R were upregulated during the hemostasis and inflammatory phase. AT_1_R mainly contributed to clotting blood, initiating inflammatory response, and benefiting epithelialization [26, 27]. Conversely, AT_2_R was assumed predominantly facilitating the differentiation of the newly formed epithelial layer, mitigates the inflammatory response, and maintained a balance between granulation and scar tissue formation at the remodeling stage. Recent studies had proved that AT_1_R antagonists like losartan relieve hypertrophic scar and keloid. Losartan reduced migration and contractile activity of human fibroblasts by down-regulating the gene expressions of collagen I, TGF-β, and monocyte chemoattractant protein-1. This, in turn, blocked the activity of myofibroblasts and the trafficking of monocytes to the scar tissues [28]. Valsartan, an AT_1_R antagonist was reported to reverse the overexpression of collagen in fibroblasts induced by Ang II [29]. The unique AT_2_R non-peptide agonist (Compound 21) had proved to prevent hypertrophic scar by reducing expression and signaling of pro-fibrotic mediators including connective TGF and TGF-β_1_. These evidences demonstrated that a compound inhibiting the pathways of AT_1_R while stimulating AT_2_R signals was desirable for treating hypertrophic scar.

**Fig. 7:**
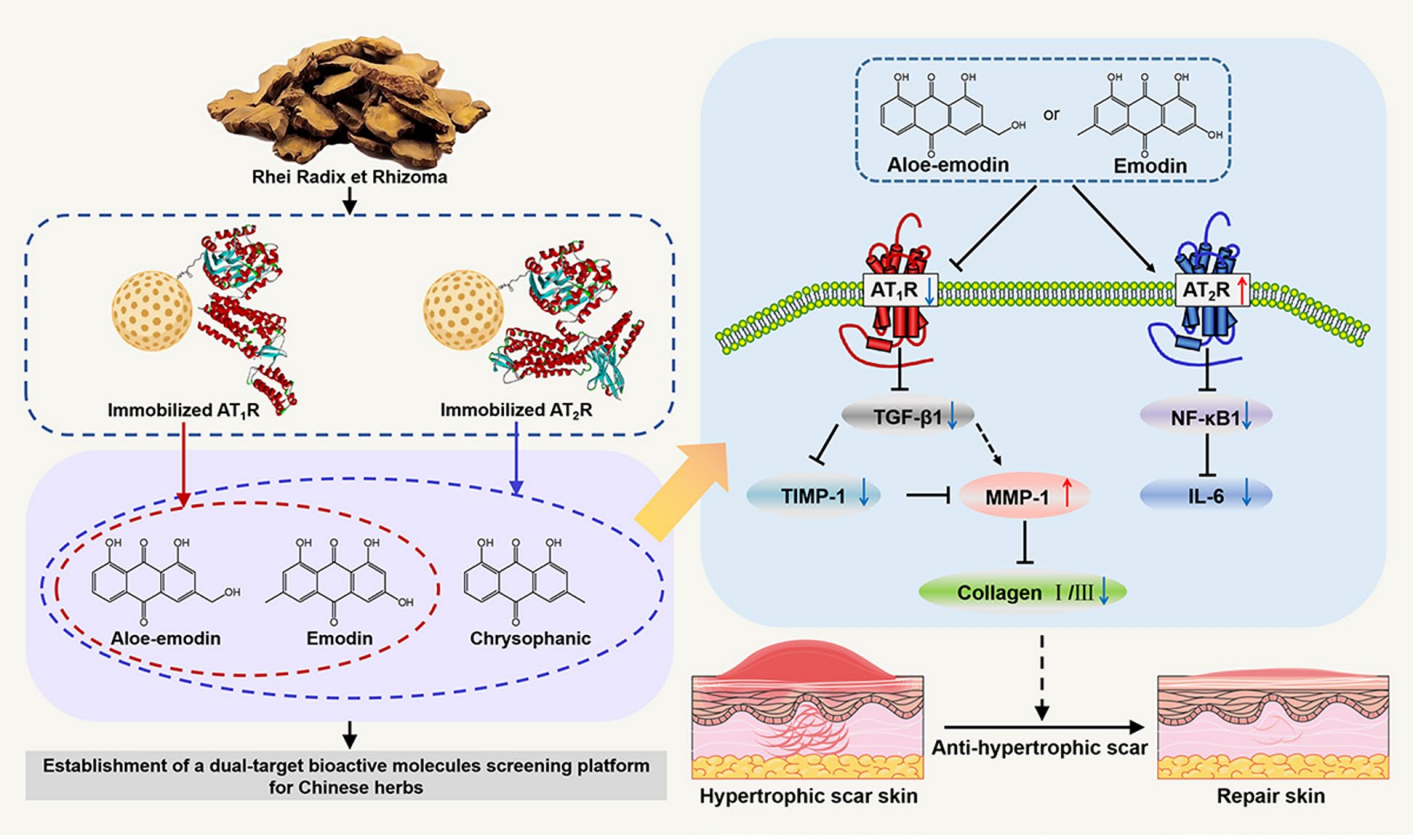
Summary of the mechanism of aloe-emodin and emodin for the treatment of cutaneous hypertrophic scar by AT_1_R and AT_2_R pathways

Rhei Radix et Rhizoma, an ancient and significant herb for the treatment of hypertrophic scar in Chinese clinical practice, was derived from the medicinal components of *Rheum palmatum* L., *Rheum tanguticum* Maxim. ex Balf., or *Rheum officinale* Baill. [30], and was recognized in the *Chinese Pharmacopoeia (2020 edition)* for its ability to address stagnation, eliminate damp heat, alleviate fire, cool blood, resolve blood stasis, and detoxify. Its main components included aloe-emodin, chrysophanol, emodin, physcion, and rhein [31]. Traditionally, powder rhubarb was mainly applied in scar treatment while anthraquinone played the key role [32]. However, there is no systematic study for bioactive compound within, so we clarified the therapeutic key substances by screening via affinity chromatography. In our current study, we screened aloe-emodin and emodin from Rhei Radix et Rhizoma as the active ingredients for the treatment of hypertrophic scar by the joint use of immobilized AT_1_R and AT_2_R. To our knowledge scope, this was a concept-of-proof study that pursues dual-target bioactive compounds from complex matrices like extract of Rhei Radix et Rhizoma. The two compounds were effective to fight hypertrophic scar in a synergistic way as they had the capacity to inhibit AT_1_R signals with simultaneous stimulation of the AT_2_R pathway. Collectively, we established a strategy for screening dual-target bioactive molecules from traditional Chinese medicine. Application of the method in the analysis of Rhei Radix et Rhizoma enabled the screening of aloe-emodin and emodin as dual-target compounds of AT_1_R and AT_2_R (Fig. 7). In vitro and in vivo tests demonstrated that the two compounds improved fibrosis by inhibiting AT_1_R whereby the expression of TGF-β1 was reduced. This prevented MMP-1 from being hydrolyzed owing to the inhibition of TIMP-1. The stimulation MMP-1 reduced the expression of collagen I/III. On the other hand, the two compounds inhibited the release of NF-кB1 and IL-6 due to the activation of AT_2_R. This was in good line with the report where emodin was demonstrated potential in hypertrophic scar treatment through the inhibition of phosphoinositide 3-kinase (PI3K)/Akt [33], the notch, and TGF-β pathways [34] in macrophages. As such, the current strategy was possible to become a universal platform for screening multi-target compounds from complex matrices when it comes to diverse diseases. Meanwhile, the pharmacological activity of the screened dual-target active ingredient from Rhei Radix et Rhizoma was superior to that of the positive control drug valsartan, thus demonstrating the potential in treating hypertrophic scar.

## Conclusions

Taking inspiration from the role of AT_1_R and AT_2_R in scar regulation, this work created a strategy for pursuing dual-target bioactive compounds from Chinese herbs to fight the ailment. The main features included: (1) except for AT_1_R, immobilized AT_2_R was firstly synthesized as a stationary phase by Halo-tag mediating bioorthogonal reaction; (2) the column containing immobilized AT_1_R and AT_2_R were comprehensively characterized to provide a platform for screening bioactive compounds binding to the two receptors from Chinese herbs; and (3) aloe-emodin and emodin were screened and confirmed as dual-target compounds blocking AT_1_R function while at the same time simulating AT_2_R anti-inflammatory pathway, thus synergistically fighting cutaneous hypertrophic scar. Taking together, aloe-emodin and emodin were possible to become candidates against hypertrophic scar. Using the stationary phases containing diverse immobilized receptors by sequential was suitable for screening dual-target bioactive compounds from complex matrices like Chinese herbs.

## Supplementary Information

Supplementary material 1.

## Data Availability

Not applicable.

## Abbreviations

AED: Adsorption energy distribution
Ang II: Angiotensin II
ANOVA: Analysis of variance
AT_1_R: Angiotensin II type 1 receptor
AT_2_R: Angiotensin II type 2 receptor
BCA: Bicinchoninic acid
DIEA: N, N-diisopropyl ethylamine
E.coli: *Escherichia coli*
GPCRs: G-protein coupled receptors
H&E: Hematoxylin-eosin
HATU: 2-(7-Azabenzotriazol-1-yl)-N,N,N′,N′-tetramethyluronium hexafluorophosphate
HPLC: High-performance liquid chromatography
HSF: Human skin fibroblasts
IL-6: Interleukin-6
LB: Luria-Bertani
MMP-1: Matrix metalloproteinase-1
NC: Nitrocellulose filter
NF-ΚB1: Nuclear factor-ΚB1
PSMF: Phenylmethanesulfonyl fluoride
RIPA: Radio immunoprecipitation assay
TGF-β1: Transforming growth factor-β1
TIMP-1: Tissue inhibitors of matrix metalloproteinase-1
TNF-α: Tumor growth factor alpha
XPS: X-ray photoelectron spectroscopy
