# Supplementary material for: A strategy for selective screening of dual-target bioactive compounds against hypertrophic scar through inhibiting angiotensin II type 1 receptor while stimulating type 2 receptor from Chinese herbs
Source: Chin Med. 2025 Jan 27;20:15. doi: 10.1186/s13020-025-01065-6 (PMC11771114; doi:10.1186/s13020-025-01065-6)
Supplement: Supplementary file 1 — Supplementary material 1. [file 13020_2025_1065_MOESM1_ESM.doc]

RESEARCH

**A strategy for selective screening of** **dual-target bioactive compounds against hypertrophic scar through inhibiting angiotensin II type 1 receptor while stimulating type 2 receptor from Chinese herbs**

Lejing Qu, Meizhi Jiao, Zilong Zhang, Yuanyuan Ou, Xue Zhao, Yajun Zhang*, Xinfeng Zhao

Key Laboratory of Resource Biology and Biotechnology in Western China, Ministry of Education, College of Life Sciences, Northwest University, Xi'an, 710069, China

*Corresponding author: Yajun Zhang

Tel.: (+86) 029 88302686

E-mail: [zhangyj@nwu.edu.cn](mailto:zhangyj@nwu.edu.cn)

**
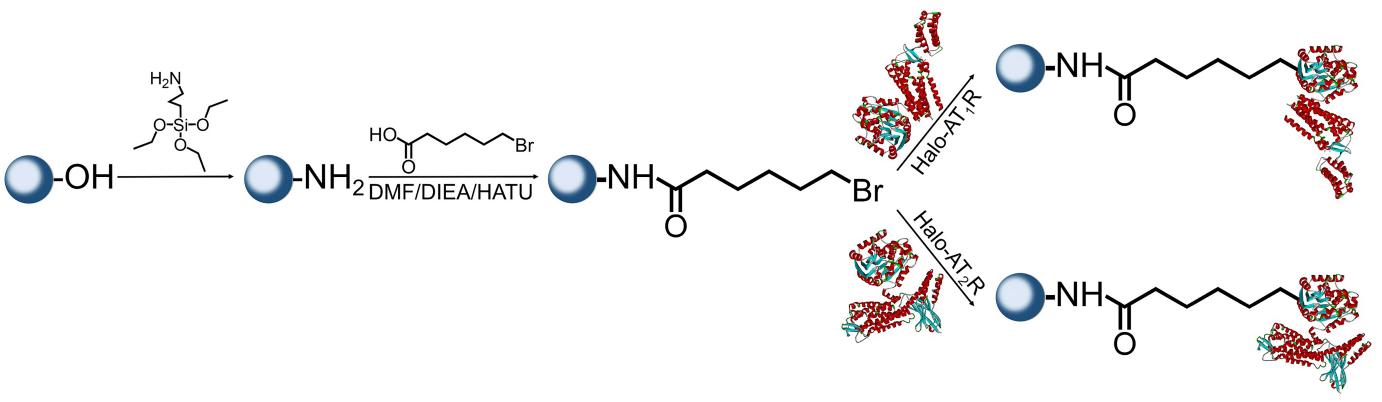
Fig. S1** Schematic diagram of the immobilization of AT_1_R and AT_2_R by the bioorthogonal reaction.

**
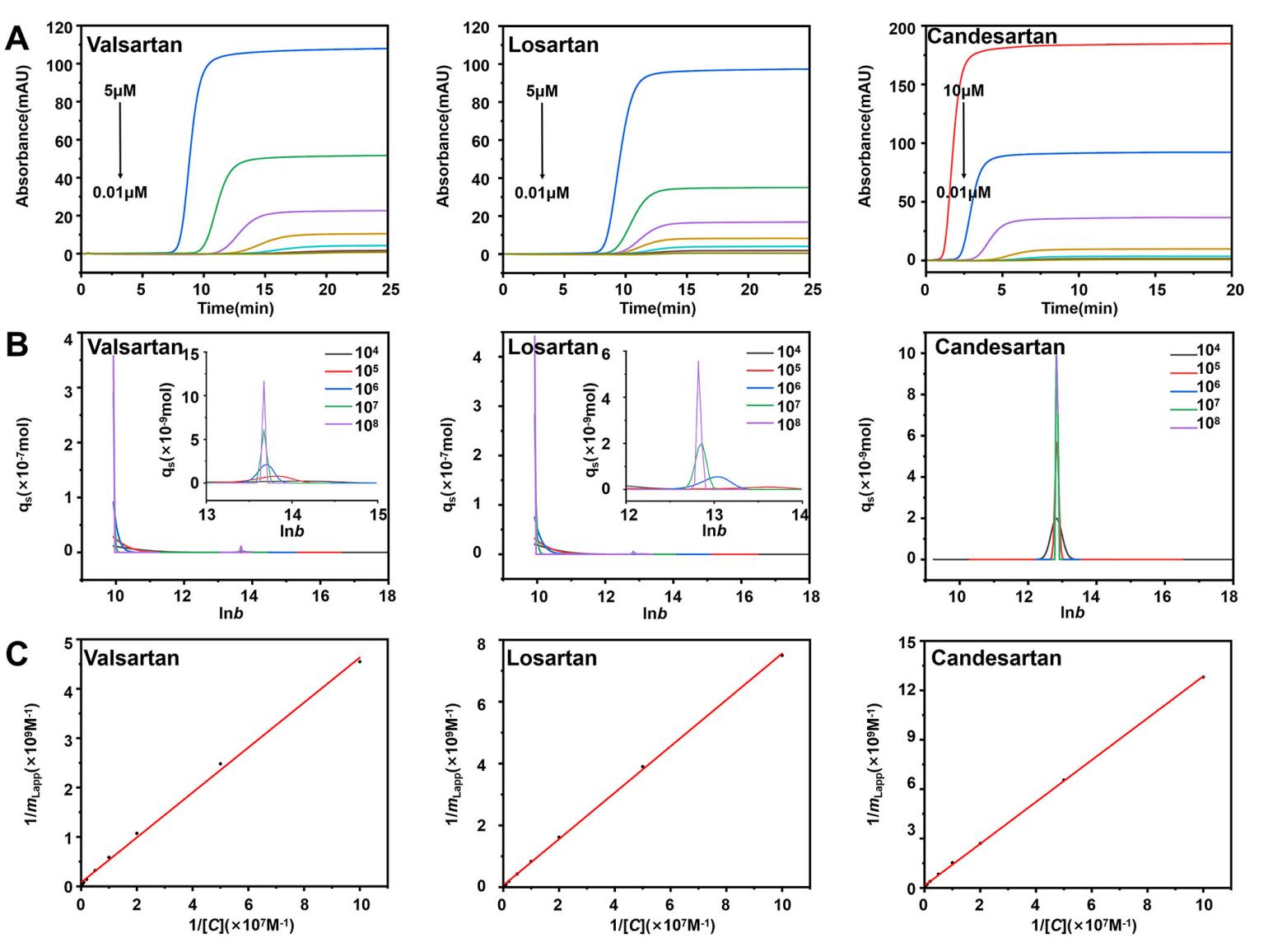
Fig. S2** The AT_1_R column in probing drug-receptor binding affinity. (A) Breakthrough curves. (B) AED analysis. (C) Plots of 1/*m*_Lapp_ versus 1/[*C*] for ligands on the AT_1_R column.

**
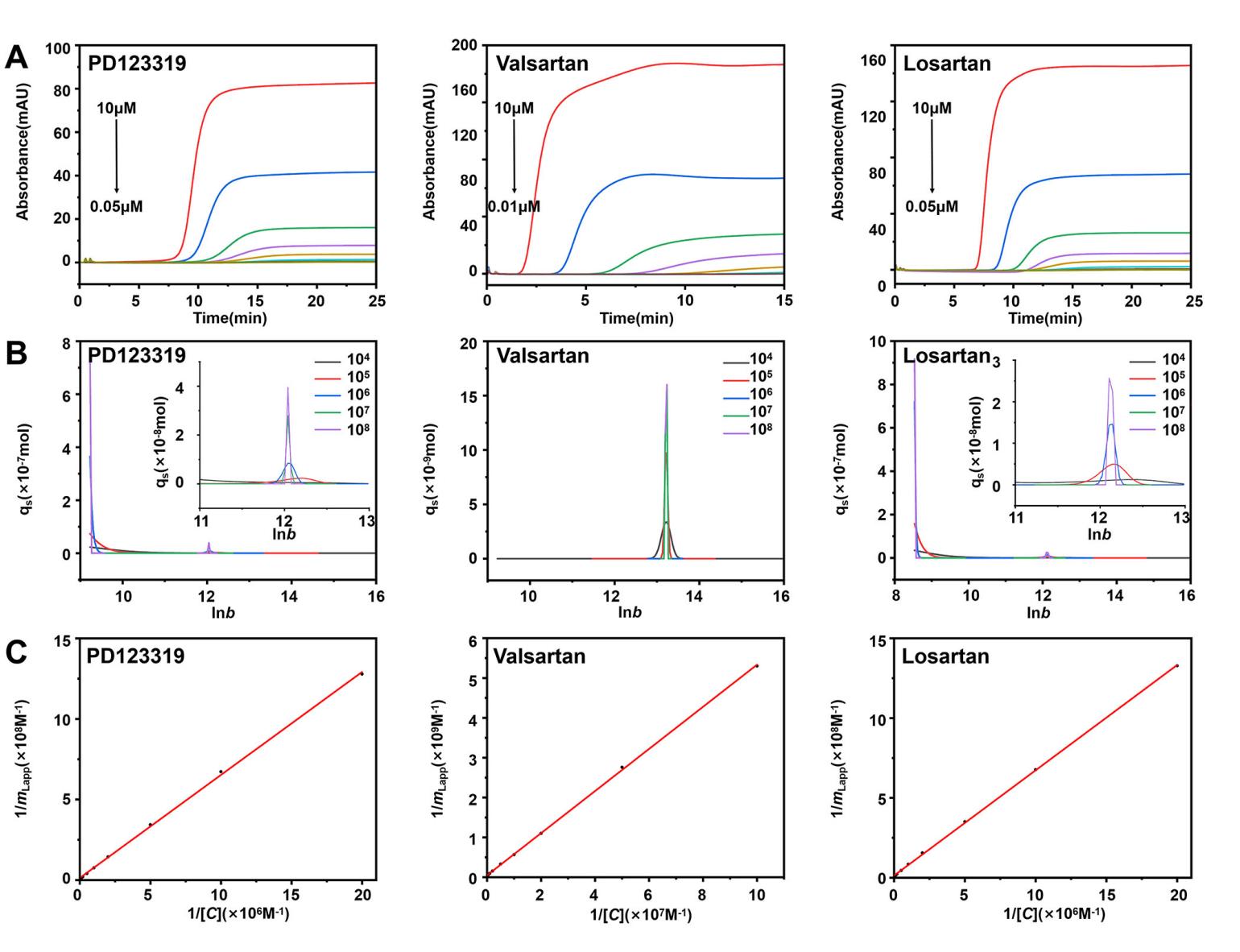
**

**Fig. S3** The AT_2_R column in probing drug-receptor binding affinity. (A) Breakthrough curves. (B) AED analysis. (C) Plots of 1/*m*_Lapp_ versus 1/[*C*] for ligands on the AT_2_R column.

**
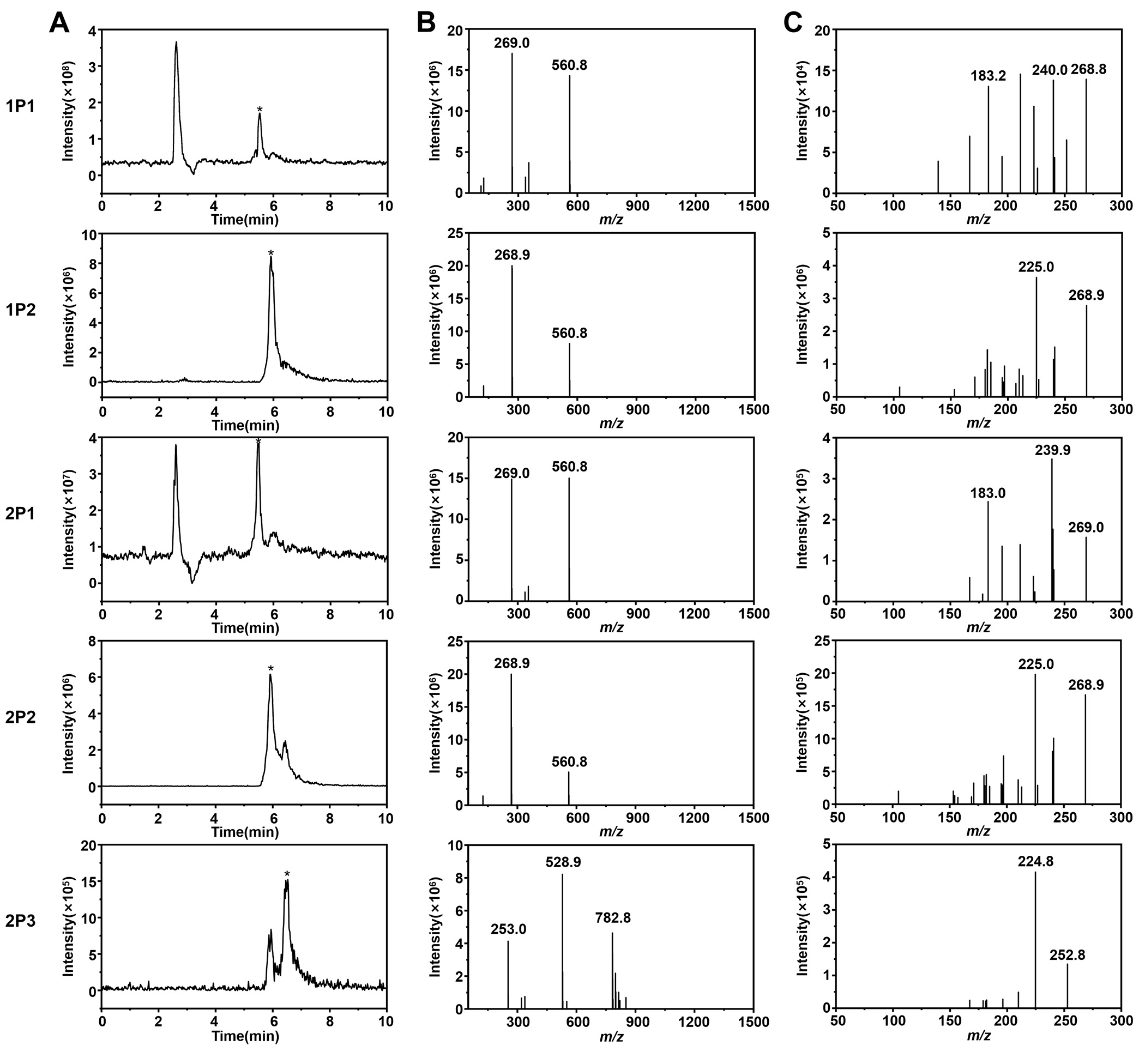
**

**Fig. S4** HPLC-MS/MS analysis of bioactive compounds in Rhei Radix et Rhizoma specifically binding to the immobilized AT_1_R or AT_2_R. (A) Total ion current spectra analyzed by HPLC-MS/MS negative mode. (B) The precursor ion spectra. (C) The product ion spectra.

**
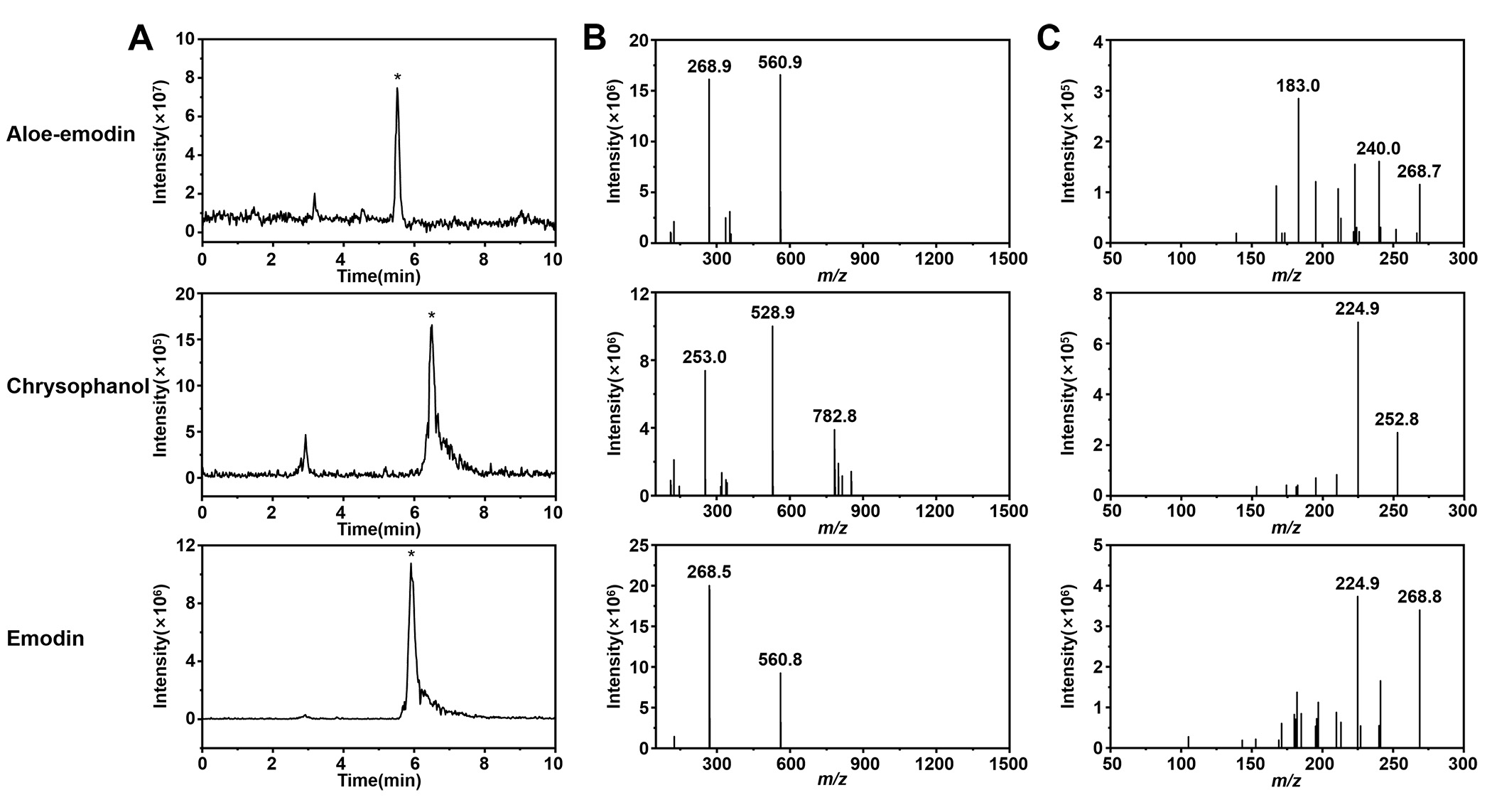
Fig. S5** HPLC-MS/MS analysis of Rhei Radix et Rhizoma standards. (A) Total ion current spectra analyzed by HPLC-MS/MS negative mode. (B) The precursor ion spectra. (C) The product ion spectra.

**Table S1** Auto-induction medium configuration method

| Reagents | Weight/Volume |
| --- | --- |
| Tryptone | 20.00 g |
| Yeast extract | 10.00 g |
| Disodium succinate | 5.40 g |
| Sodium citrate | 0.30 g |
| D-Glucose | 0.50 g |
| α-Lactose monohydrate | 2.00 g |
| Disodium hydrogen phosphate dodecahydrate | 3.55 g |
| Ammonium chloride | 2.68 g |
| Magnesium sulfate | 0.52 g |
| Ferric chloride | 0.03 g |
| Glycerol | 25.00 mL |
| Reverse osmosis water | 1000.00 mL |

**Table S2** Analysis of element distribution on silica gel

| Elements | Atomic% | | | | |
| --- | --- | --- | --- | --- | --- |
|  | silica gel | aminopropyl silica gel | 6-hexabromohexanoic acid coated gel | immobilized AT_1_R silica gel | immobilized AT_2_R silica gel |
| C 1s | 33.38±0.02 | 38.33±0.08 | 44.41±0.35 | 51.47±0.02 | 48.15±0.34 |
| O 1s | 43.67±0.03 | 38.37±0.11 | 34.21±0.31 | 28.56±0.20 | 30.74±0.45 |
| Si 2p | 22.95±0.01 | 20.49±0.13 | 18.18±0.27 | 14.08±0.01 | 15.52±0.34 |
| N 1s | 0.00 | 2.81±0.06 | 3.00±0.10 | 5.84±0.19 | 5.58±0.15 |
| Br 3d | 0.00 | 0.00 | 0.2±0.01 | 0.05 | 0.01 |

**Table S3** Results of the ANOVA overall difference test between groups for protein levels in HSF cells

|  | Sun of Squares | Degrees of Freedom | Mean Square | F | Sig. |
| --- | --- | --- | --- | --- | --- |
| AT_1_R/GAPDH | 15.098 | 4 | 3.774 | 33.514 | 0.000 |
| AT_2_R/GAPDH | 1.976 | 4 | 0.494 | 17.671 | 0.000 |
| TGF-β1/GAPDH | 5.925 | 4 | 1.481 | 24.401 | 0.000 |
| MMP-1/TIMP-1 | 12.126 | 4 | 3.032 | 73.206 | 0.000 |
| Collagen Ⅰ /GAPDH | 3.733 | 4 | 0.933 | 7.473 | 0.005 |
| Collagen Ⅲ /GAPDH | 1.222 | 4 | 0.306 | 66.59 | 0.000 |
| NF-κB1/GAPDH | 5.827 | 4 | 1.457 | 17.106 | 0.000 |
| IL-6/GAPDH | 4.220 | 4 | 1.055 | 9.519 | 0.002 |

**Table S4** Results of the ANOVA overall difference test between groups for protein levels in rabbit

|  | Sun of Squares | Degrees of Freedom | Mean Square | F | Sig. |
| --- | --- | --- | --- | --- | --- |
| AT_1_R/GAPDH | 1.498 | 4 | 0.374 | 12.286 | 0.001 |
| AT_2_R/GAPDH | 4.335 | 4 | 1.084 | 37.018 | 0.000 |
| TGF-β1/GAPDH | 1.599 | 4 | 0.4 | 10.434 | 0.001 |
| MMP-1/TIMP-1 | 4.221 | 4 | 1.055 | 20.759 | 0.000 |
| Collagen Ⅰ /GAPDH | 2.884 | 4 | 0.721 | 14.64 | 0.000 |
| Collagen Ⅲ /GAPDH | 1.84 | 4 | 0.46 | 11.518 | 0.001 |
